# Supplementary material for: Chromothripsis during telomere crisis is independent of NHEJ, and consistent with a replicative origin
Source: Genome Res. 2019 May;29(5):737–49. doi: 10.1101/gr.240705.118 (PMC6499312; doi:10.1101/gr.240705.118)
Supplement: Supplemental Material [file supp_gr.240705.118_Supplemental_file_1.zip › contigs/annotated_contigs/DB69/contig.2.DB69_length_275_mean_cov_17.8109090909.docx]

**DB69_length_275_mean_cov_17.8109090909**

```
AAAATTTGGTATTTTGTGTGGTGGATGAGAATGTAGGCTTCTATTCTGCCCTCTTATTCTGTCTCTGCCTTAAATTTTTTAGATTCAGG
 >chr9:66470737-66470955 - E=3e-100
GAGTATATTTGCAGGTTTATTACATGGATATATTGTGATGCTGAGCAATGTGGGATATTATTGATCCCATCACCAAGGTAGTGAGTATA

TATTGTGGGATATAATTGATCCCATCACCTAGGCAGT|TAGCAGAACATTTATAC|TTTCTCACCCCTTCCCCTCCTCCTTCCCTCCTT
                                                       >chrY:20529943-20529986 - E=2e-0
CATCTAGTAG8
```
